# Editorial: “Purinergic Signaling 2020: The State-of-The-Art Commented by the Members of the Italian Purine Club”

**DOI:** 10.3389/fphar.2021.768923

**Published:** 2021-09-14

**Authors:** Francisco Ciruela, Kjell Fuxe, Peter Illes, Henning Ulrich, Francesco Caciagli

**Affiliations:** ^1^ Pharmacology Unit, Department of Pathology and Experimental Therapeutics, School of Medicine and Health Sciences, Institute of Neurosciences, University of Barcelona, L’Hospitalet de Llobregat, Spain; ^2^ Neuropharmacology & Pain Group, Neuroscience Program, Bellvitge Institute for Biomedical Research, IL’Hospitalet de Llobregat, Spain; ^3^ Department of Neuroscience, Karolinska Institutet, Biomedicum, Stockholm, Sweden; ^4^ Rudolf Boehm Institute for Pharmacology and Toxicology, University of Leipzig, Leipzig, Germany; ^5^ International Collaborative Centre on Big Science Plan for Purinergic Signalling, Chengdu University of Traditional Chinese Medicine, Chengdu, China; ^6^ Department of Biochemistry, Institute of Chemistry, University of São Paulo, São Paulo, Brazil; ^7^ Department of Medical, Oral and Biotechnological Sciences, University of Chieti-Pescara, Chieti, Italy

**Keywords:** purines, purinergic signaling, purinergic enzymes, purinergic receptor, purinergic receptor signaling

The “purinergic signaling” term was coined in 1972 by Geoffrey Burnstock Burnstock et al. after demonstrating that adenosine 5’-triphosphate (ATP) is a transmitter in nonadrenergic, noncholinergic inhibitory nerves innervating the guinea-pig taenia coli (Burnstock et al., 1966). This signaling system, which is ubiquitously expressed in every organ and system of the body, comprises various ecto-, soluble and intracellularly localized enzymes, nucleoside transporters, and G protein-coupled and ligand-gated cation channel receptors. Through the purinergic signaling system cells can maintain basal adenine and guanine-based purines at certain steady-state levels, thereby contributing to preserve the purines-dependent cellular homeostasis.

Extracellular levels of nucleotides and nucleosides may fluctuate enormously while being degraded by the action of several ectonucleotidases, which rapidly metabolize ATP to ADP, AMP, and adenosine. These extracellular purines are in constant equilibrium with their intracellular counterparts through cell surface transporters, thus balancing the purine content within both compartments. Purinergic receptors are subdivided into P1, for nucleosides, and P2 for nucleotides. While P1 purinoceptors are G protein-coupled receptors, P2 are both G protein-coupled and ligand-gated cation channel receptors, thus complementing each other in their signaling. Often, the same cell concurrently expresses different subtypes of P1 and P2 receptors, which allows the integration of purinergic transmission into short- and long-term signaling events. Overall, the coordinated function of purinergic enzymes, transporters, and receptors allows the cells to exquisitely harmonize their purinergic signaling. Accordingly, deciphering the precise molecular interplay between the different purinergic signaling partners, in health and disease, will propel the therapeutic use of purine-based compounds in numerous diseases, including cancer, metabolic and CNS disorders.

In this research topic, escorted by the Italian Chapter of Purine Club, an overview of the purinergic signaling field is provided through 40 articles written by about 200 authors. This successful compilation of manuscripts contains 23 opinions, five minireviews, five brief research reports, four perspectives, one general commentary, one review and one original research paper. The opinion articles collect the opinions from top leading experts in the field, thus promoting the debate of controversial scientific aspects as well as assessing future perspectives of clinical applications of purines. Indeed, purinergic receptors constitute a hot topic within these opinion papers. For instance, the instrumental role of P2Y_12_ receptors in the microglial response to neuropathological lesions (Lin et al.) or the antagonistic functions of P2Y_2_ and P2X_7_ receptors in neurodegenerative diseases (Glaser et al.) are highlighted. The structural features and potential therapeutic opportunities for P2X_3_ receptor ligands are discussed based on the fact that these receptors are confined to nociceptive neurons, thus making them an attractive target for the management of inflammatory, neuropathic, and visceral pain states and chronic and refractory chronic cough (Spinaci et al.). The Janus-face of P2X_7_ receptors in amyotrophic lateral sclerosis is debated (Volonté et al.), thus establishing the importance of peripheral *vs*. central clues that drive motor neuron and neuromuscular impairment, paralysis, and finally death in amyotrophic lateral sclerosis. Similarly, P1 receptors are further considered within these opinion papers. The role of purines, in general (Magni and Ceruti), and adenosine receptors, in particular (Coppi et al.; Luongo et al.), within the pathophysiology of chronic neuropathic pain is nicely updated, thus highlighting the recent research revealing the central role of adenosine A_3_ receptors. Also, the potential use of adenosine A_2A_ receptor as a novel target for Alzheimer’s disease (Merighi et al.) is discussed. Certainly, adenosine A_1_ receptor partial agonists and positive allosteric modulators may serve to overcome the clinical drawbacks found in the pharmacotherapeutic usefulness of the activation of this receptor in several diseases (Vincenzi et al.). In addition, P1 and P2 purinergic receptors as valuable targets to stimulate myelin repair in the pathophysiology of multiple sclerosis are discussed (Lecca et al.). Next, the pharmacological modulation of the adenosinergic system to manage and counteract obesity and its related comorbidities is further commented (D’Antongiovanni et al.), thus encouraging the development of novel adenosine receptor ligands. Finally, the possible interactions between adenosine and kynurenic acid, two well-known neuromodulators, in the pathophysiology of schizophrenia are critically analyzed (Beggiato et al.).

Guanosine, considered an orphan neuromodulator (Di Liberto et al.), also attracted the attention of the experts of the field although the precise mechanism of its action is still unknown. While adenosine receptors have been involved in some guanosine-mediated physiological effects, the identification of the adenosinergic target of guanosine, if any, is still on the way (Massari et al.). The potential role of guanosine in the skeletal muscle-central nervous system axis communication through guanosine-stuffed exosomes delivered to neurons in the brain constitutes a hot topic (Pietrangelo). Finally, the role and prospective use of guanine-based purines in the management of cancer and major depressive (Almeida et al.) and aging-dependent (Di Iorio et al.) disorders is discussed.

The limitations of using inhibitors and/or antibodies against CD39 and CD73 as immunotherapeutic tools is discussed within the framework of a purinergic system cross-talk, which includes the different nucleotides and nucleosides, enzymes and receptors, and the extracellular environment (Battastini et al.). Thus, the notion of considering the purinergic system as a whole is reflected by the purinome concept, which provides an integrative framework to understand those purinergic alterations that may have important pathological consequences, as observed in glioblastoma multiforme, the most common/lethal human brain tumor (Giuliani et al.), or even in the dynamics of disease progression in sepsis (Leite-Aguiar et al.). Another example is the use of humanized mouse models to investigate the role of purinergic signaling in the inflammatory immune disorder (Sluyter and Watson). The evidence for a cross-talk between cytosolic 5’-nucleotidases and AMP-activated protein kinase is also analyzed (Camici et al.). Next, the importance of different forms of CD73 as prognostic biomarker of tumor progression or as predictive biomarker of responses to anticancer therapies in cancer patients is argued (Turiello et al.). Finally, and to conclude with the section of “Opinion,” a historical overview of the discovery and characterization of the novel dinucleotide uridine adenosine tetraphosphate (Up4A) within the cardiovascular system is considered (Zhou and Matsumoto).

Four perspective manuscripts put the eye on some relevant purinergic signaling issues. For instance, Barresi et al. provide an up-to-date highlight of the recent findings and future perspectives in the field of adenosine A_2B_ receptor orthosteric and allosteric ligands (Barresi et al.). The role of the striatal-enriched protein tyrosine phosphatase in adenosine A_2A_ receptor-mediated effects in the central nervous system is contemplated by Maria Rosaria Domenici and collaborators (Domenici et al.). The role of adenosine receptor-containing heteromers balancing the opioid and dopaminergic transmission in the striatum (Borroto-Escuela et al.) and biasing signaling (Franco et al.) is further discussed. Finally, a general commentary manuscript revises the recent publication in Cell (Prescott et al., 2020)reporting a novel role of P2Y_1_ receptors in vagal sensory neurons and their involvement in initiating a series of airway defense reflexes that guard the airways from external stimuli (Liu et al.).

Subsequently, one review and five minireviews highlight diverse aspects of purinergic signaling. For instance, the role of ectonucleotidases in acute and chronic inflammation is nicely reviewed by Anna Lisa Giuliani and collaborators (Giuliani et al.) reporting that ectonucleotidases play a major role in chronic inflammation by setting the balance between pro-inflammatory nucleotides and anti-inflammatory adenosine. Next, the current knowledge on the structure and functions of purinergic P2 receptors in mechanotransduction in health and disease is outlined (Kong et al.). An integrative picture of the molecular mechanisms leading to changes in feeding behaviour within hypothalamic neurons following purinergic receptor activation is presented by Caruso and collaborators (Caruso et al.). The role of extracellular ATP and adenosine as potent extracellular signaling molecules in the retina is also summarized (Ye et al.). Marta Lombardi *et al*. discuss the current knowledge on the role of ATP in the biogenesis and dynamics of extracellular vesicles (Lombardi et al.). Finally, recent developments on positron emission tomography tracers for imaging adenosine A_2A_ receptors and their applications in the diagnosis and treatment of adenosine-related diseases are discussed (Sun et al.).

The research topic contains a series of original research manuscripts covering important aspects of purinergic signaling. Thus, five brief research reports investigate the role of guanosine on human neuroblastoma cell differentiation (Belluardo et al.), the contribution of plasmin generation in the proangiogenic effect of adenosine A_2A_ receptor upon activation (Valls et al.), the effects of a P2X7 receptor agonist in cell viability and cortico-striatal synaptic transmission in experimental models of Huntington’s Disease (Martire et al.), the expression pattern and activation profile of P2Y receptors in TGF-β1-mediated cardiac fibrosis (Tian et al.) and the impact of vascular purinergic signaling in erythrocyte induce endothelial injury in type 2 diabetes (Mahdi et al.). Finally, an original research article investigates the role of Ecto-5’-nucleotidase (CD73) in the allergic airway inflammation upon sensitization, thus mice lacking CD73 show an exacerbated allergic airway inflammation (Caiazzo et al.).

By compiling this set of manuscripts, we aim to build up a scientific framework helping to propel the discovery of novel purine-based pharmacological tools as well as potential diagnostic or prognostic biomarkers within the purinergic field, thus paving the way for personalized medicine. Overall, this research topic constitutes a virtual roundtable for the scientific purinergic community to share the more recent findings and innovative ideas concerning the use of purines as new pharmacological tools. Finally, we would like to dedicate this research topic to the memory of Prof. Geoffrey Burnstock, the father of the purinergic signaling field, who peacefully passed away on June 3rd, 2020.

